# Contribution of nuclear and mitochondrial gene mutations in mitochondrial encephalopathy, lactic acidosis, and stroke-like episodes (MELAS) syndrome

**DOI:** 10.1007/s00415-020-10390-9

**Published:** 2021-01-23

**Authors:** Sanjiban Chakrabarty, Periyasamy Govindaraj, Bindu Parayil Sankaran, Madhu Nagappa, Shama Prasada Kabekkodu, Pradyumna Jayaram, Sandeep Mallya, Sekar Deepha, J. N. Jessiena Ponmalar, Hanumanthapura R. Arivinda, Angamuthu Kanikannan Meena, Rajan Kumar Jha, Sanjib Sinha, Narayanappa Gayathri, Arun B. Taly, Kumarasamy Thangaraj, Kapaettu Satyamoorthy

**Affiliations:** 1 Department of Cell and Molecular Biology, Manipal School of Life Sciences, Manipal Academy of Higher Education, Manipal, India; 2 Department of Neuropathology, National Institute of Mental Health and Neurosciences (NIMHANS), Bangalore, India; 3 Department of Neurology, National Institute of Mental Health and Neurosciences (NIMHANS), Bangalore, India; 4 Neuromuscular Laboratory, Neurobiology Research Centre, National Institute of Mental Health and Neurosciences (NIMHANS), Bangalore, India; 5 Department of Bioinformatics, Manipal School of Life Sciences, Manipal Academy of Higher Education, Manipal, India; 6 Department of Neuroimaging and Interventional Radiology, National Institute of Mental Health and Neurosciences (NIMHANS), Bangalore, India; 7 Department of Neurology, Nizam’s Institute Medical Sciences (NIMS), Hyderabad, India; 8 CSIR-Centre for Cellular and Molecular Biology, Hyderabad, India; 9 Institute of Bioinformatics, International Tech Park, Bangalore, India; 10 Manipal Academy of Higher Education, Manipal, India; 11 Genetic Metabolic Disorders Service, Children’s Hospital At Westmead, Sydney, NSW Australia; 12 Centre for DNA Fingerprinting and Diagnostics, Hyderabad, India; 13 Discipline of Child and Adolescent Health, Faculty of Medicine and Health, The University of Sydney, Sydney, NSW Australia

**Keywords:** MELAS, Mutations, Nuclear genome, mtDNA, CNV

## Abstract

**Background:**

Mitochondrial disorders are clinically complex and have highly variable phenotypes among all inherited disorders. Mutations in mitochon drial DNA (mtDNA) and nuclear genome or both have been reported in mitochondrial diseases suggesting common pathophysiological pathways. Considering the clinical heterogeneity of mitochondrial encephalopathy, lactic acidosis and stroke-like episodes (MELAS) phenotype including focal neurological deficits, it is important to look beyond mitochondrial gene mutation.

**Methods:**

The clinical, histopathological, biochemical analysis for OXPHOS enzyme activity, and electron microscopic, and neuroimaging analysis was performed to diagnose 11 patients with MELAS syndrome with a multisystem presentation. In addition, whole exome sequencing (WES) and whole mitochondrial genome sequencing were performed to identify nuclear and mitochondrial mutations.

**Results:**

Analysis of whole mtDNA sequence identified classical pathogenic mutation m.3243A > G in seven out of 11 patients. Exome sequencing identified pathogenic mutation in several nuclear genes associated with mitochondrial encephalopathy, sensorineural hearing loss, diabetes, epilepsy, seizure and cardiomyopathy (*POLG*, *DGUOK*, *SUCLG2*, *TRNT1*, *LOXHD1*, *KCNQ1*, *KCNQ2*, *NEUROD1*, *MYH7*) that may contribute to classical mitochondrial disease phenotype alone or in combination with m.3243A > G mutation.

**Conclusion:**

Individuals with MELAS exhibit clinical phenotypes with varying degree of severity affecting multiple systems including auditory, visual, cardiovascular, endocrine, and nervous system. This is the first report to show that nuclear genetic factors influence the clinical outcomes/manifestations of MELAS subjects alone or in combination with m.3243A > G mutation.

**Electronic supplementary material:**

The online version of this article (10.1007/s00415-020-10390-9) contains supplementary material, which is available to authorized users.

## Introduction

Defective mitochondrial function contributes to childhood and adult neurometabolic disorders affecting multiple organs with a projected global prevalence of 1 in 5000 [1]. Patients with mitochondrial disease manifests with a heterogeneous clinical phenotype, varying age of onset and different grades of severity [2]. Previous studies have identified more than 1500 nuclear genome encoded proteins participating in various mitochondrial function [3]. Due to this dual nature of genetic control, mutations in either nuclear or mitochondrial genomes or both may contribute to mitochondrial dysfunction [4, 5]. Mitochondrial diseases are classified based on mutations in either the mitochondrial or the nuclear genome affecting intraorganellar gene expression and function [6].

Nuclear gene defects identified in mitochondrial diseases are primarily responsible for mtDNA replication, transcription and translation, oxidative phosphorylation (OXPHOS) and biogenesis of mtDNA [7]. The other nuclear genes contribute to various mitochondrial disorders involved in nucleoside transport, salvage or synthesis; and maintenance of balanced mitochondrial deoxyribonucleoside triphosphates (dNTP) pool [8]. MELAS is a clinically complex early onset multi-organ mitochondrial disorder with diverse phenotype and varying degree of severity [9, 10]. These patients clinically present with encephalomyopathy, lactic acidosis, and stroke-like episodes and show evidence of ragged red fibers (RRF) on muscle biopsy. In addition, there are a wide range of other clinical symptoms which include diabetes mellitus, cardiomyopathy, migraine, epilepsy, cerebellar ataxia, impaired vision, and hearing loss in varying combination [11]. Majority of these patients harbor mutation in the *MT-TL1* gene coding for the tRNALeu (UUR). The m.3243A > G mutation has been reported in 80% of MELAS patients affecting tRNALeu (UUR) aminoacylation and mitochondrial translation of OXPHOS protein subunits [12–14]. However, recent study suggests that patients with m.3243A > G mutation exhibiting the vast clinical heterogeneity underpinning the necessity to interrogate nuclear genome for better understanding of complex mitochondrial disorders [15]. This study aimed to sequence the whole mitochondrial genome and the whole exome of clinically characterized MELAS patients and identify mutations in both nuclear and mitochondrial genome and their contribution to its heterogeneous phenotype.

## Methods

### Patients

A total of eleven patients with clinically suspected MELAS syndrome referred to Department of Neurology, National Institute of Mental Health and Neurosciences, Bangalore, India; and Department of Neurology, Nizam’s Institute of Medical Sciences, Hyderabad, India, were included in this study. All patients had comprehensive clinical evaluation, neurological and ophthalmological examination, biochemical tests, and magnetic resonance imaging (MRI). Routine histopathological examination of skeletal muscle was done in 10 patients. Institutional Ethical Committee (IEC) clearance was obtained from the participating institute (NIMHANS/IEC (BS & NS DIV.) 8th meeting/2017). Patients were enrolled after obtaining the written informed consent.

### Pathological studies

Fresh skeletal muscle biopsy of the patients was used for histopathology, electron microscopy (EM) and enzyme histochemistry. Histological [Hematoxylin and Eosin (H&E), modified Gomori trichrome (MGT) staining) and histochemical [cytochrome c oxidase (COX), succinate dehydrogenase (SDH) and combined COX/SDH] analysis was performed using standard protocol [16]. A small fragment of the muscle fixed in 3% glutaraldehyde was subjected to mitochondrial ultra-structural analysis using transmission electron microscopy (JEOL200X, Japan) by established protocols described elsewhere [17, 18].

### Biochemical analysis

Tissue homogenates from frozen muscle biopsies were extracted as described earlier [19] and used for the enzyme assay of the four mitochondrial respiratory chain complexes (I, II, III and IV) using spectrophotometry in six patients [19, 20].

### Whole mitochondria genome sequencing

DNA was isolated from both peripheral blood and skeletal muscle tissue using the method described earlier [21]. Whole mitochondrial genome was amplified by using 24 sets of primers to generate overlapping fragments [22]. PCR amplicons were electrophoresed using 2% agarose gel, purified with Exo-SAP and sequenced using BigDye Terminator ready reaction kit (Applied Biosystems, Foster City, USA). Post PCR purification of amplified products were performed by ethanol precipitation and dissolved in Hi-Di formamide. PCR amplicons were sequenced using ABI 3730 automated DNA analyzer (Applied Biosystems). The sequences were aligned with revised Cambridge reference sequence (rCRS) (NC_012920) [23, 24] using Sequence Analysis and AutoAssembler tools. All the mismatched nucleotide sequences were obtained and compared with 300 healthy controls, and further compared with Mitomap database (http://www.mitomap.org). Haplogroup analysis was performed using phylotree [(www.phylotree.org; mtDNA tree Build 17 (18 Feb 2016)]. Further, m.3243A > G heteroplasmic levels of the patients and their available maternal relatives were assessed using the PCR-RFLP method as described earlier [25].

### Whole exome sequencing

Whole exome sequencing (WES) was performed using Ion AmpliSeq™ Exome RDY Kit 4 × 2 kit (ThermoFisher Scientific, Waltham, MA, USA) according to manufacturer’s protocol. Library preparation of ultra-high multiplex PCR enriched whole exome amplicons was carried out using Ion AmpliSeq™ Library Kit Plus (Thermo Fisher Scientific) as per the manufacturer’s instructions. Each sample was barcoded using Ion Express Barcode Adapter 1–16 kit (Thermo Fisher Scientific). Quantification of each library was performed on Agilent Bioanalyzer 2100 using Agilent high sensitivity (HS) DNA kit (Agilent Technologies, Santa Clara, CA, USA). WES was performed using Ion Proton PI v3 chip in Ion Proton next generation sequencing platform (Thermo Fisher Scientific) as per the manufacturer’s instruction.

### In silico analysis

Sequence read alignment to human reference genome sequence (Hg19) and variant calling were carried out using Torrent Variant Caller v5.12 (TVC) in Torrent Suite software (Thermo Fisher Scientific). After removal of low-quality reads, variants were detected using Ion TVC using optimized parameters (AmpliSeq Designer, Thermo Fisher Scientific) for low-frequency variant detection with minimal false-positive calls. Variant filter used the technical parameters which include 1) variant quality score, 2) variant coverage, and 3) variant allele frequency. Coverage analysis was done using Coverage Analysis Plugin software v5.12. Visual inspection of read alignment and variants called were performed using Integrative Genomic Viewer (IGV v2.8.2) software [26] to investigate for strand biases, homopolymer length and sequencing errors. Variant caller format (VCF) generated by variant caller plug-in was further analyzed using Ion Reporter software v5.12 (Thermo Fisher Scientific) for variant annotation and filtering.

Silent and known germline variants were removed based on population frequency using dbSNP, NHLBI Exome Sequencing Project (https://esp.gs.washington.edu/EVS/) and Exome Aggregation Consortium (http://exac.broadinstitute.org/) (variants with more than 1% minor allele frequency, MAF). Variant prioritization was performed to select variants that alter protein function; these included nonsenses, splice site, coding indel, or missense variants.

### Variant analysis

VCF files for each exome datasets from MELAS patients were uploaded to Ingenuity variant analysis (IVA, Qiagen Bioinformatics, USA) program [27]. Each exome dataset was rigorously filtered to identify pathogenic variants based on quality, MAF, deleteriousness, inheritance patterns, conservation and its association with clinical phenotype of each MELAS patients. Additionally, we analyzed heterozygous SNVs identified in MELAS patients as per the American College of Medical Genetics and Genomics (ACMG) guidelines and the prediction algorithms used by Varsome, the allele frequencies in the Genome Aggregation Database (GnomAD), Exome Aggregation Consortium-ExAC (http://exac.broadinstitute.org/) and multiple in silico tools for prediction of pathogenicity which includes DANN, DEOGEN2, EIGEN, FATHMM-MKL, M-CAP, MVP, MutationAssessor, MutationTaster, PrimateAI, REVEL and SIFT [28]. In addition, we have compared the mutations/ prioritized variants with our internal database consisting of 51 whole exome data of other mitochondrial disorder patients.

### Copy number variation analysis

Copy number variation (CNV) analysis of whole exome sequencing data of MELAS patients was performed using EXCAVATOR2 v1.1.2 [29]. EXCAVATOR2 employs off target reads aligning outside targeted regions for improved detection of CNVs when compared with other exome-based CNV callers such as XHMM and CoNIFER. Each MELAS exome and control BAM files were analyzed through the EXCAVATOR2 pipeline using the standard settings.

## Results

### Clinical presentation of MELAS patients

**Fig. 1:**
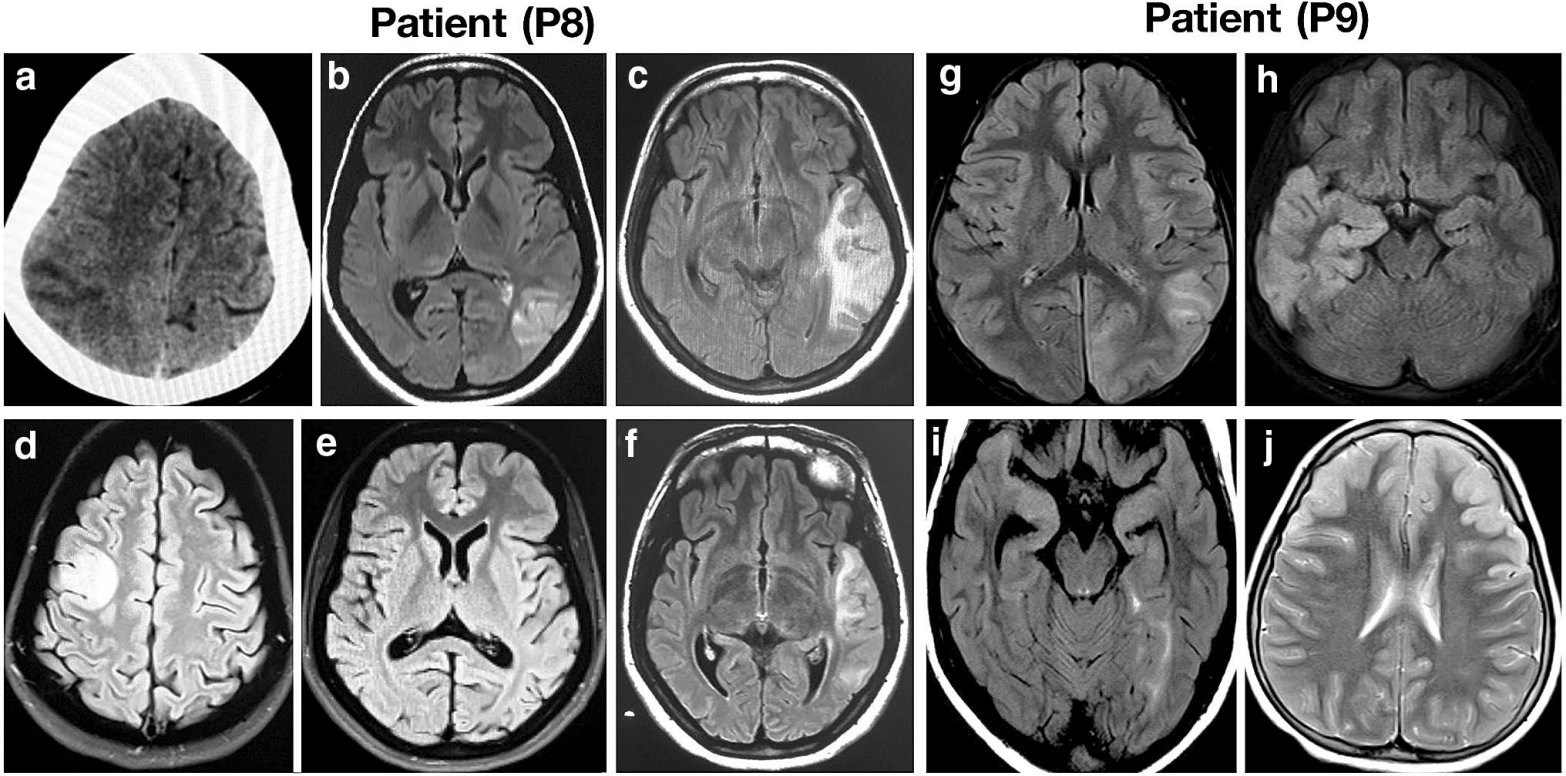
CT and MRI brain of MELAS patients with nuclear gene mutations. Axial sections of CT and MRI of brain show shifting cortical-based signal changes that do not conform any arterial territory in patient P8 (A–F). CT brain shows an ill-defined focal hypodensity in the left parietal region in 2008 (**a**), Brain MRI shows focal wedge-shaped hyperintensity in the left temporal region resembling an ‘infarct’ in 2010 (**b**, **c**), focal hyperintensity in the right frontal region in 2012, while the lesion seen in the left temporal region in 2010 has resolved completely (**d**, **e**) and recurrence of focal hyperintensity in the left temporal region in 2014 (**f**). Axial sections of MRI of brain show shifting hyperintensities involving the cortex that do not conform an arterial territory in patient P9 (**g**–**j**). Brain MRI shows focal hyperintensity in the left parieto-occipital region in September 2012 (**g**), focal hyperintensity in the right temporal region in November 2012 (**h**), focal hyperintensity in left medial temporal region in January 2013 (**i**) and hyperintensity in bilateral frontal region in August 2016 (**j**)

**Fig. 2:**
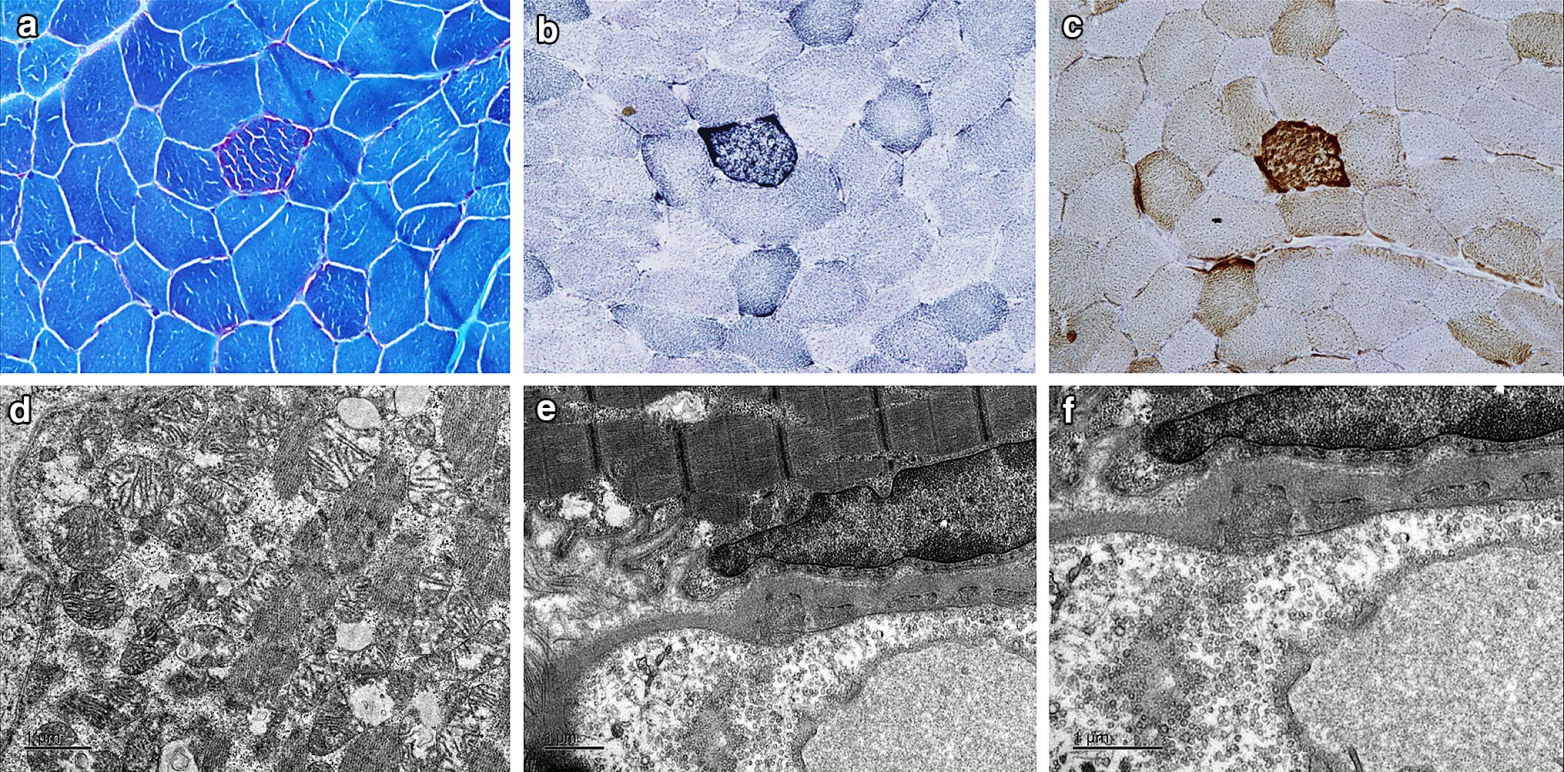
Light and electron microscopic features of muscle biopsy from a patient negative for m.3243A > G mutation showing evidence of mitochondrial insult. **a** ragged red fibers (MGT); **b** Ragged blue fibers (SDH); **c** No COX-deficient fiber (COX- SDH); *d* Aggregates of mitochondria with altered cristae in the sub-sarcolemmal region; **e**, **f** Increased pinocytic vesicles in the endothelial cells of blood vessels

Clinical presentation of 11 patients having MELAS (6 males and 5 females) is summarized in Table 1. The mean age at presentation was 20 $\pm$ 11.3 years (age range 7–45 years) and mean age at onset was 14.2 $\pm$ 10.6 years (age range 6–41 years). Majority of the patients presented with seizures, status epilepticus, hearing impairment and had elevated lactate. Magnetic resonance imaging (MRI) of brain showed stroke-like lesions predominantly in parieto-occipital cortex (Fig. 1) in eight patients (Table 1). Histopathology analysis of muscle biopsy showed ragged red fibers (RRF) and ragged blue fibers (RBF) (Fig. 2a–c) in five patients including one patient who was negative for m.3243A > G. However, there were no COX-deficient fibers in any of these cases. Electron microscopy analysis done in five patients, whose muscle biopsy did not reveal any diagnostic pathology on light microscope, showed sub-sarcolemmal aggregation of mitochondria of varying size and altered cristae pattern (Fig. 2d). In addition, increased pinocytic vesicles were noted in the endothelial cells of blood vessels (Fig. 2e–f). Respiratory chain complex assay was performed in six patients and revealed isolated complex I (P10, P11) and IV deficiency (P2, P9) in two patients each and multiple complex deficiency in two patients (P4, P5) (Table 1).

**Table 1:** Clinical features, MRI, pathology, biochemical abnormalities and mtDNA mutations in MELAS patients

Patient ID	Age (Year)/ gender	Age at onset (Years)	Consanguinity and Family history	Clinical features	Neuroimaging	Serum Lactate (mg/dL) Normal<20mg/dl	Muscle biopsy	RCC enzyme assay	mtDNA pathogenic mutation	Heteroplasmic level (% mutant) for m.3243A>G	Mitochondrial haplogroup
P1	16/M	13	Non-consanguineous No family history of neurological illness,	Hemicranial headache, altered sensorium, seizures, stroke-like episodes, left ventricular hypertrophy	T2/FLAIR hyperintense signal changes in left parieto-occipital cortex	32	RRF and RBF	NA	NC_012920.1:m.3243A>G	Blood: 50% Muscle: 90% *Mother: 10% *Brother: 10% (Both were asymptomatic)	R31b
P2	45/M	41	Consanguineous Mother had deafness, younger brother had similar history and expired during one episode of status epilepticus (not evaluated) Both paternal and maternal relatives have history of diabetes mellitus	Status epilepticus at onset, recurrent stroke-like episodes, deep venous thrombosis, During follow-up, the patient developed hearing impairment, neuropathy, diabetes mellitus, and cognitive and behavioral problems. Prolonged P100 latency on VEP	Shifting stroke-like lesions in the parieto-tempero-occipital regions on MRI. Bilateral basal ganglia calcification and right frontal small calcified lesion on CT scan,	32	RRF and RBF	Complex IV deficiency	NC_012920.1:m.3243A>G NC_012920.1:m.4317A>G	Blood: 30% Muscle: 87%	M35b2
P3	10/F	6	Non-consanguineous Muscle cramp, migraine, lipoma and thyroid cancer in mother Maternal grandmother had young onset diabetes, impaired hearing, stroke and cancer and died at the age of 49 years	Recurrent seizures, status epilepticus, migraine like episodes, visual disturbances, failure to thrive, dysphagia, short stature, proximal muscle weakness and hyporeflexia. Died at the age of 12 years	Stroke like lesions involving both hemispheres and eventually leading to diffuse cerebral and cerebellar atrophy. Bilateral basal ganglia calcification	97	NA	NA	NC_012920.1:m.3243A>G	Blood: 70% *Mother: 58%	M7c1a2a1
P4	11/M	6	Non-consanguineous Maternal grandmother had short stature. Neonatal death in elder sibling. History of diabetes mellitus in multiple family members on maternal side	Developmental delay, retarded physical growth, stroke-like episodes, status epilepticus, epilepsia partialis continua, impaired hearing and vision, and cognitive decline	Multiple small stroke-like lesions, cerebral atrophy, bilateral basal ganglia calcification on CT scan	52	RRF and RBF	Multiple complex (I+IV) deficiencies	NC_012920.1:m.3243A>G	Blood: 75%	U7a3b
P5	12/M	11	Non-consanguineous No family history of neurological illness	Recurrent stroke-like episodes, seizures, migraine, status epilepticus, constipation, visual impairment and Valproate induced hepatotoxicity	Shifting parieto-occipital cortical lesions involving both sides	49	RRF and RBF	Multiple complex (I+IV) deficiencies	NC_012920.1:m.3243A>G	Blood: 52%	M53b
P6	26/F	20	Consanguineous	Seizure, cognitive decline	NA	NA	No diagnostic pathology	NA	NC_012920.1:m.3243A>G	Blood: 23%	NA
P7	20/M	6	Consanguineous	Ataxia, seizures, diminished visual acuity	NA	NA	No diagnostic pathology	NA	NC_012920.1:m.3243A>G	Blood: 27%	NA
P8	20/F	16	Non-consanguineous	Headache with vomiting, focal motor seizures involving both sides, psychosis, aphasia, impaired vision	Shifting cortical lesions involving multiple lobes both sides	35	No diagnostic pathology	NA	No pathogenic mutation	Blood: Nil Muscle: Nil	NA
P9	7/F	6	Non-consanguineous Hypothyroidism and young onset diabetes in father, seizures and diabetes on paternal side and hypothyroidism in maternal grandmother	Headache, recurrent seizures associated with motor deficits involving both sides, behavioral abnormalities, aphasia. Four years in the course of illness patient was detected to have Autoimmune encephalitis: NMDA positive versus NMDA-MELAS overlap	Shifting cortical lesions involving parieto-tempero-occipital regions both sides	34.1	No diagnostic pathology	Complex IV deficiency	No pathogenic mutation	Blood: Nil Muscle: Nil	M2a1a
P10	33/M	23	Non-consanguineous Mother had history of migraine and cardiac arrhythmia requiring pacemaker	Impaired vision with optic atrophy, impaired hearing, recurrent migraine, alternating hemiplegia, focal motor seizures, epilepsia partialis continua, behavioral abnormalities	Shifting cortical abnormality involving right parieto-occipital region once and bilateral frontal parietal temporal occipital region later	43.9	RRF and RBF	Complex I deficiency	No pathogenic mutation	Blood: Nil Muscle: Nil	HV13
P11	19/F	9	Non-consanguineous	Status epilepticus, focal seizures with generalization, cognitive decline, mild ataxia, Prolonged P100 latency on VEP	Shifting cortical lesions involving different lobes, predominantly right side	35.2	No diagnostic pathology	Complex I deficiency	NC_012920.1:m.13135G>A (possible hypertrophic cardiomyopathy susceptible factor)	Blood: Nil Muscle: Nil	M18a

M-Male, F-Female, RRF- Ragged red fibers, RBF-Ragged blue fibers, NMDA- N-methyl-D-aspartate, VEP-Visual evoked potential, NA-Not available, * Blood DNA

### Mutations in mitochondrial genome

Whole mitochondrial genome sequencing analysis identified pathogenic mutation m.3243A > G of *MT-TL1* gene in seven patients, and none in the remaining four patients (Table 1). In addition, one patient (P2) with m.3243A > G was also carrying m.4317A > G in *MT-TI* gene. Another patient (P11) negative to m.3243A > G was carrying m.13135G > A in *MT-ND5* gene that is reported as possible hypertrophic cardiomyopathy susceptible factor. Phylogenetic analysis showed that the patients were from different haplogroup background (Table 1).

### Mutations in nuclear genome

Whole exome sequencing analysis of eleven patients generated an average of 34 million mapped reads with more than 92% reads on target in all MELAS patients in Ion Proton NGS platform (Supplementary Table 1). The mean per base depth of coverage for the exome consensus coding sequence was 97-fold, with 93% of bases covered more than 20-fold (Supplementary Table 1). Patients with the MELAS phenotype present with wide spectrum of clinical features ranging from seizures, stroke-like episodes, ataxia, sensorineural hearing loss, diabetes and epilepsy. Phenotype-driven analysis and prioritization of causal mutations in each MELAS patient was performed using Ingenuity variant analysis software. Phenotype-driven analysis identified deleterious protein coding mutations in nuclear genes with known mitochondrial function previously reported in mitochondrial disorders (*POLG*, *DGUOK, SUCLG2, TRNT1*) (Table 2).

**Table 2:** Nuclear gene mutations identified in MELAS syndrome subjects

Gene symbol	Function	In MitoCarta	Transcript change	Protein change	Literature cited	Zygosity	ACMG Classification	*In silico* analysis^#^	Patients
*POLG*	mtDNA replication	Yes	NM_001126131.1(POLG):c.2243G>C	p.(Trp748Ser)	Tzoulis et al., 2006	Homozygous	Pathogenic (PS1, PM1, PP2, PP3, PP5)	Pathogenic	2 (P6, P7)
			NM_001126131.1(POLG):c.3559C>T	p.(Arg1187Trp)	Reichenbach. et al., 2006	Heterozygous	Uncertain significance (PM1, PP2, PP3)	Pathogenic	3 (P1, P3, P5)
*DGUOK*	dNTP metabolism	Yes	NM_080916.2(DGUOK):c.468del	p.(Phe156Leufs*45)	Present study	Homozygous	Pathogenic (PVS1, PM2, PP3)	Pathogenic	2 (P10, P11)
*SUCLG2*	dNTP metabolism	Yes	NM_003848.3(SUCLG2):c.235G>T	p.(Glu79*)	Present study	Heterozygous	Uncertain significance (PM2, PP3)	Pathogenic	3 (P1, P3, P5)
*TRNT1*	post-transcriptional modification of tRNAs	Yes	NM_182916.2(TRNT1):c.947_948insG	p.(Glu318Argfs*11)	Present study	Homozygous	Pathogenic (PVS1, PM2, PP3)	Pathogenic	1 (P1)
*LOXHD1*	Normal function of hair cells in the inner ear	No	NM_144612.6(LOXHD1):c.4690C>T NM_144612.6(LOXHD1):c.2054G>A	p.(Leu1564Phe) p.(Arg685His)	Present study	Compound Heterozygous	Uncertain significance (PM2, PP3)	Pathogenic	1 (P2)
			NM_144612.6(LOXHD1):c.5813G>A NM_144612.6(LOXHD1):c.1191G>T	p.(Arg1938His) p.(Trp397Cys)	Present study	Compound Heterozygous	Uncertain significance (PM2, PP3)	Pathogenic	1 (P4)
*KCNQ1*	Potassium channel protein active in heart muscle	No	NM_000218.3(KCNQ1):c.575G>A	p.(Arg192His)	Present study	Heterozygous	Likely pathogenic (PM1, PM2, PP2, PP3)	Pathogenic	1 (P9)
*KCNQ2*	Potassium channel protein active in neurons	No	NM_172107.4(KCNQ2):c.598C>A	p.(Leu200Met)	Present study	Heterozygous	Likely pathogenic (PM1, PM2, PM5, PP2, PP3)	Pathogenic	1 (P9)
*NEUROD1*	Transcription factor regulates insulin gene expression	No	NM_002500.4(NEUROD1):c.175G>C	p.(Glu59Gln)	Chapla et al., 2015	Heterozygous	Uncertain significance (PP3)	Pathogenic	1 (P2)
*MYH7*	Codes for beta (β)-myosin heavy chain protein contractions of cardiac muscle	No	NM_000257.4(MYH7):c.1013T>C	p.(Val338Ala)	Present study	Heterozygous	Likely pathogenic (PM1, PM2, PP2, PP3)	Pathogenic	1 (P8)

*POLG* (DNA polymerase subunit gamma) is solely responsible for replication of mitochondrial DNA [30]. *POLG* mutation has been reported in both autosomal dominant and autosomal recessive mitochondrial disorders [31]. In our study, we identified two patients (P6 and P7) with homozygous missense *POLG* mutation (p.Trp748Ser), previously reported in patients with progressive neurological disorder (Table 2) [31, 32]. Additionally, we identified heterozygous *POLG* mutation (p.Arg1187Trp) in three patients (P1, P3 and P5), which was previously reported in two cases with mitochondrial respiratory chain diseases [33, 34].

*DGUOK* encodes enzyme deoxyguanosine kinase, which is essential for maintaining building blocks of mitochondrial DNA. Mutation in *DGUOK* is well documented in patients with mitochondrial DNA depletion syndrome leading to neurological conditions with liver dysfunction [35]. We identified homozygous frameshift deletion in *DGUOK* (p.Phe156LeufsTer45) in two patients (P10 and P11; Table 2). The heterozygous mutation in *SUCLG2*, c.235G > T (p.Glu79*) has been identified in three MELAS patients (P1, P3 and P5). In addition, homozygous frameshift insertion in *TRNT1* (p.Glu318Argfs*11) was observed in one patient (P1) (Table 2).

MELAS patients in our study showed a wide range of clinical presentation including hearing loss, diabetes, epilepsy, seizures and impaired vision. We identified heterozygous missense mutation in *NEUROD1* (p.Glu59Gln) in one patient (P2), which was previously reported to be associated with maturity-onset diabetes of the young (MODY) phenotype in Indian population [36, 37]. Compound heterozygous mutation in *LOXHD1* c.4690C > T (p.Leu1564Phe) c.2054G > A (p.Arg685His) and c.5813G > A (p.Arg1938His), c.1191G > T (p.Trp397Cys) was observed in two patients (P2, P4) with hearing impairment. Additionally, we identified heterozygous *KCNQ1* mutation c.575G > A (p.Arg192His) and heterozygous *KCNQ2* mutation c.598C > A (p.Leu200Met) in one MELAS patient (P9) (Table 2).

### Influence of nuclear gene mutations on m.3243A > G

We looked at the control of nuclear gene mutations on the clinical expression of m.3243A > G and found patient P2 harbor variants in two genes namely *LOXHD1* (p.Leu1564Phe, p.Arg685His) and *NEUROD1* (p.Glu59Gln) which is reported to be associated with non-syndromic hearing loss and maturity-onset diabetes, respectively. This is likely that these variations could have contributed to deafness in patient and the mother, and diabetes mellitus in multiple family members (Table 1). Another patient P3 harbored variants in two genes namely *POLG* (p.Arg1187Trp) and *SUCLG2* (p.Glu79*) reported to be associated with mitochondrial respiratory chain diseases and mtDNA depletion, impaired respiratory complex subunits and mitochondrial encephalopathy, respectively. These gene variants may be modifiers for the severe clinical course and premature death.

### Copy number variants identified in MELAS syndrome

CNV analysis of MELAS exome datasets identified recurrent copy number amplification in 2q34 (*ERBB4*), 4q12 (*ADGRL3*), 5p12 (*HCN1*), 8q23 (*CSMD3*), 11p14 (*LRRC4C*) and 14q11.2 (*FOXG1*). We observed copy number gain in 5p12 (*HCN1*) and 14q11.2 (*FOXG1*) in multiple MELAS individuals (Supplementary Table 2).

## Discussion

Mitochondrial disorders have complex clinical phenotypes and thus pose a significant challenge in the clinical diagnosis. These are caused by genetic mutations in mitochondrial DNA, nuclear DNA or both [39, 40]. We have performed a detailed clinical, biochemical, imaging, and molecular analysis of 11 patients with suspected MELAS syndrome. Whole mitochondrial genome sequencing identified a pathogenic m.3243A > G mutation in seven patients. Recent studies have identified nuclear gene mutations in suspected MELAS patients and other classical mitochondrial phenotypes highlighting the need to screen nuclear gene mutations and function for improved diagnosis of mitochondrial disorders [38–42]. To achieve this, we have performed WES of 11 MELAS patients and identified pathogenic mutation in nuclear encoded genes associated with mitochondrial encephalopathy (*POLG*, *DGUOK, SUCLG2, TRNT1*), sensorineural hearing loss, seizures, epileptic encephalopathy, and cardiomyopathy (*LOXHD1*, *KCNQ1*, *KCNQ2* and *MYH7*). Mutations in *POLG* and *DGUOK* have been previously implicated in mitochondrial disorders with common phenotype such as mitochondrial respiratory complex defect, mitochondrial DNA depletion and encephalopathy [42, 43].

Homozygous *POLG* mutation c.2243G > C (p.Trp748Ser) identified in two patients with m.3243A > G (P6 and P7) was previously reported in ataxia patients of European origin [31]. *POLG* mutation is also associated with multiple mtDNA deletion syndrome [31]. However, histopathology of muscle in these two patients (P6 and P7) in our study did not show any abnormality as reported previously in European patients with this mutation [32]. Additionally, both P6 and P7 did not have classical MELAS phenotype based on the available clinical data. It is interesting to note that sequencing of mitochondrial genome along with exome sequencing identified these double pathogenic mutations in two patients (P6 and P7). Hence, our observations indicate that pathogenic mutation in both nuclear (*POLG*) and mitochondrial (*MT‐TL1*, m.3243A > G) in two patients (P6 and P7) corroborates the role of dual genetic mutations in MELAS phenotype. In contrast to our observation, a study identified *POLG1* mutations in MELAS-like phenotype but negative for common mtDNA mutations [42]. Another study reported heterozygous *POLG1* variants p.Arg627Gln and p.Gly848Ser in a patient with stroke-like episodes typical of MELAS phenotype negative for m.3243A > G [44].

*DGUOK* mutation (p.Phe156LeufsTer45) was seen in two patients negative for m.3243A > G (P10 and P11). Interestingly, patient (P10) showed RRF, RBF and aggregates of mitochondria with altered cristae in the sub-sarcolemmal region (Fig. 2a–d). Functional role of *DGUOK* in mitochondrial DNA maintenance is well established [35]. Mutation in *DGUOK* has been previously reported in patients with neurological abnormality [43]. Mutation in *TRNT1* and *SUCLA2* has been implicated in patients with mitochondrial encephalopathy [45, 46].

*SUCLG2* encodes a GTP-specific beta subunit of succinate-CoA ligase (SUCL), an important enzyme in TCA cycle, and primarily expressed in liver [47]. Homozygous knockout model of *SUCLG2* in mice showed recessive lethality [48]. Patients with SUCL deficiency showed mtDNA depletion, impaired respiratory complex subunits and mitochondrial encephalomyopathy [47–49]. Mutation in *SUCLG2* has not been reported so far in any patients with mitochondrial disorder. In this study, we report for the first time, a heterozygous mutation in *SUCLG2* gene, which introduces a nonsense codon in the ATP-grasp_2 domain. The glutamate residue in the 79 position in the *SUCLG2* protein is responsible for substrate specificity. However, a detailed functional analysis is needed to establish the role of *SUCLG2* nonsense mutation (p.Glu79*) in the mitochondrial dysfunction identified in our study. *TRNT1* (CCA-adding transfer RNA nucleotidyl transferase) is the key enzyme which plays an important role in cytosolic and mitochondrial tRNA modification and protein translation [50]. We identified a homozygous frameshift insertion in *TRNT1* (p.Glu318Argfs*11) in one patient (P1). A previous study reported wide range of phenotypes with *TRNT1* mutations both in childhood and adult onset retinitis pigmentosa, encephalopathy, hepatosplenomegaly and pancreatic insufficiency [46].

In our study, patients with sensorineural hearing loss, seizures, epileptic encephalopathy and cardiomyopathy showed mutations in *LOXHD1*, *KCNQ1*, *KCNQ2* and *MYH7* genes. These may contribute to the severity of diverse clinical phenotype of MELAS syndrome alone or in combination with mitochondrial mutations. *LOXHD1* mutation has been previously reported in patients with non-syndromic hearing loss (NSHL) [51, 52]. Mutations in both *KCNQ1* and *KCNQ2* identified in our study were previously reported as pathogenic in the ClinVar database. Autosomal dominant mutation in *KCNQ1* causes congenital long-QT syndrome and cardiac arrhythmia [53]. *KCNQ2*, a brain-specific voltage-gated potassium-channel subunit, has been implicated in patients with epileptic encephalopathy and neonatal‐onset seizures [54]. In this study, the patient (P9) carrying *KCNQ2* mutation was positive for NMDAR (N-methyl-D-aspartate receptor) antibodies. A previous case report has shown the association of anti-NMDAR antibodies in MELAS syndrome with m.3243A > G mutation [55]. Autosomal dominant *MYH7* mutation has been reported in patients with hypertrophic cardiomyopathy [56]. We observed one patient (P8) with *MYH7* mutation which was identified as likely pathogenic mutation according to the ACMG guidelines and ClinVar database (Table 2). Most of the patients showed early onset in our cohort. The patients with m.3243A > G mutations harboring nuclear gene mutations showed varying clinical features (Supplementary Table 3). Hence, nuclear gene mutations may act as modifiers in the disease phenotype. Another interesting observation is that the patients with m.3243A > G negative showed mutations in *DGUOK, KCNQ1, KCNQ2* and *MYH7.* Further, functional validation will elucidate the role of these genes in MELAS phenotype.

MELAS syndrome patients with predominant neurodegenerative symptoms may gradually develop wide spectrum of endocrine disorders including diabetes. We have identified *NEUROD1* missense mutation (p.Glu59Gln) in one MELAS patient with diabetes. *NEUROD1* (p.Glu59Gln) mutation identified in our study has been previously reported in maturity-onset diabetes in an Indian cohort [36, 37]. Copy number variation analysis of exome sequencing data identified recurrent copy number gains in chromosomes 5p12 and 14q11.2 which harbors *HCN1* and *FOXG1* which has been previously reported in epilepsy, a clinical phenotype commonly observed in MELAS patients [57, 58]. Hence, these CNVs could contribute to the diverse phenotypes of MELAS. Studies have shown advances in the diagnostic pathways for mitochondrial diseases and ‘genetic first approach’ is preferred [59–61]. In this study, we identified nuclear mutations in MELAS patients who are negative for m.3243A > G mutation. Biopsy may not be required in patient with characteristic clinical syndrome feature harboring m.3243A > G mutation; however, patients with atypical feature and who are negative for m.3243A > G mutation require MRI, muscle biopsy and RCC assay are useful and complementary to aid for genetic diagnosis.

## Conclusion

Mitochondrial diseases are challenging to diagnose due to their wide spectrum of clinical features and genetic heterogeneity. Previous studies have highlighted the contribution of nuclear gene mutations in mitochondrial disorders. This study identified both nuclear and mtDNA mutations in clinically established MELAS patients. Our study demonstrated that the contribution of nuclear genetic background may influence the clinical heterogeneity in m.3243A > G‐related classical mitochondrial disease. This has far-reaching implications which opens the possibility of identifying new mechanisms in well-characterized larger patient cohort and pedigree analysis. A detailed evaluation of these novel interactions between nuclear genetic factors and m.3243A > G may facilitate development of more accurate tools for improved diagnosis, prognosis and better genetic counseling of patients with mitochondrial disorders. Limited number of MELAS cases evaluated in this study is the shortcoming; however, it opens up promising avenues for a larger patient cohort.

## Electronic supplementary material

Below is the link to the electronic supplementary material.

Electronic supplementary material 1 (DOCX 1081 kb)

## Data Availability

The data that support the findings of this study are available from the corresponding author upon request.
