# Supplementary material for: Contribution of nuclear and mitochondrial gene mutations in mitochondrial encephalopathy, lactic acidosis, and stroke-like episodes (MELAS) syndrome
Source: J Neurol. 2021 Jan 23;268(6):2192–207. doi: 10.1007/s00415-020-10390-9 (PMC8179915; doi:10.1007/s00415-020-10390-9)
Supplement: Supplementary file 1 — Electronic supplementary material 1 (DOCX 1081 kb) [file 415_2020_10390_MOESM1_ESM.docx]

**Supplementary data**

**Supplementary Table 1: Summary of MELAS patients exome sequence data**

| Patient ID | Mapped reads | On target (%) | Mean depth | Uniformity (%) |
| --- | --- | --- | --- | --- |
| P1 | 44,156,415 | 93.44 | 124.5 | 94.45 |
| P2 | 47,283,725 | 92.30 | 131.1 | 94.49 |
| P3 | 46,310,596 | 93.23 | 117.2 | 89.70 |
| P4 | 32,574,215 | 93.46 | 91.83 | 89.37 |
| P5 | 34,804,540 | 91.55 | 95.07 | 88.68 |
| P6 | 17,480,584 | 90.08 | 59.56 | 93.55 |
| P7 | 27,820,966 | 87.73 | 76.55 | 94.38 |
| P8 | 48,277,319 | 93.87 | 144.9 | 95.49 |
| P9 | 39,804,407 | 93.36 | 118.4 | 95.03 |
| P10 | 15,425,511 | 93.82 | 46.23 | 92.64 |
| P11 | 18,926,359 | 93.92 | 56.3 | 94.00 |

**Supplementary Table 2: Recurrent CNVs identified in MELAS patient’s exome data.**

| Chromosomal  location | Size  (kb) | Cytoband | Reference | Gene  Symbol | Copy  Number | Patients ID | Known phenotype |
| --- | --- | --- | --- | --- | --- | --- | --- |
| chr2:211562948-215214346 | 3651.4 | 2q34 | Rashidi-Nezhad et al., 2012 | *ERBB4* | Gain | P4, P9, P10 | Neurodegenerative disorder |
| chr4:58316638- 67975729 | 9659.1 | 4q12-q13.2 | Novel | *ADGRL3* | Gain | P1, P2, P4, P5 P9, P10 | - |
| chr5:43764715-46396454 | 2631.7 | 5p12-p11 | Poduri, 2014 | *HCN1* | Gain | P2, P4, P5, P11 | Epilepsy |
| chr8:110534938- 115929297 | 5394.4 | 8q23 | Floris et al., 2008 | *CSMD3* | Gain | P1, P2, P5, P9, P11 | Autism |
| chr11:37041061- 43098058 | 6056.9 | 11p14-p11 | Kaminsky et al., 2011 | *LRRC4C* | Gain | P2, P8, P9, P11 | Intellectual disability |
| chr14:25223628-30255228 | 5031.6 | 14q11.2-32.33 | Seltzer et al., 2014 | *FOXG1* | Gain | P1, P2, P4, P5, P10, P11 | Epilepsy |

**Reference**:

Rashidi-Nezhad A, Parvaneh N, Farzanfar F, Azimi C, Harewood L, Akrami SM, Reymond A (2012). 2q34-qter duplication and 4q34.2-qter deletion in a patient with developmental delay. Eur J Med Genet. 55:203‐210.

Poduri, A (2014). HCN1 Gain-Of-Function Mutations - A New Cause of Epileptic Encephalopathy. Epilepsy Curr 14: 348–349.

Floris C, Rassu S, Boccone L, Gasperini D, Cao A, Crisponi L(2008). Two patients with balanced translocations and autistic disorder: CSMD3 as a candidate gene for autism found in their common 8q23 breakpoint area. Eur J Hum Genet. 16:696‐704.

Kaminsky EB, Kaul V, Paschall J, Church DM, Bunke B, Kunig D, Moreno-De-Luca D, Moreno-De-Luca A, Mulle JG, Warren ST, Richard G, Compton JG, Fuller AE, Gliem TJ, Huang S, Collinson MN, Beal SJ, Ackley T, Pickering DL, Golden DM, Aston E, Whitby H, Shetty S, Rossi MR, Rudd MK, South ST, Brothman AR, Sanger WG, Iyer RK, Crolla JA, Thorland EC, Aradhya S, Ledbetter DH, Martin CL (2011). An evidence-based approach to establish the functional and clinical significance of copy number variants in intellectual and developmental disabilities. Genet Med. 13:777-784.

Seltzer LE, Ma M, Ahmed S, Bertrand M, Dobyns WB, Wheless J, Paciorkowski AR (2014). Epilepsy and outcome in FOXG1-related disorders. Epilepsia 55: 1292–1300.

**Supplementary Table 3: List of mtDNA and nuclear gene mutations in MELAS patients.**

**
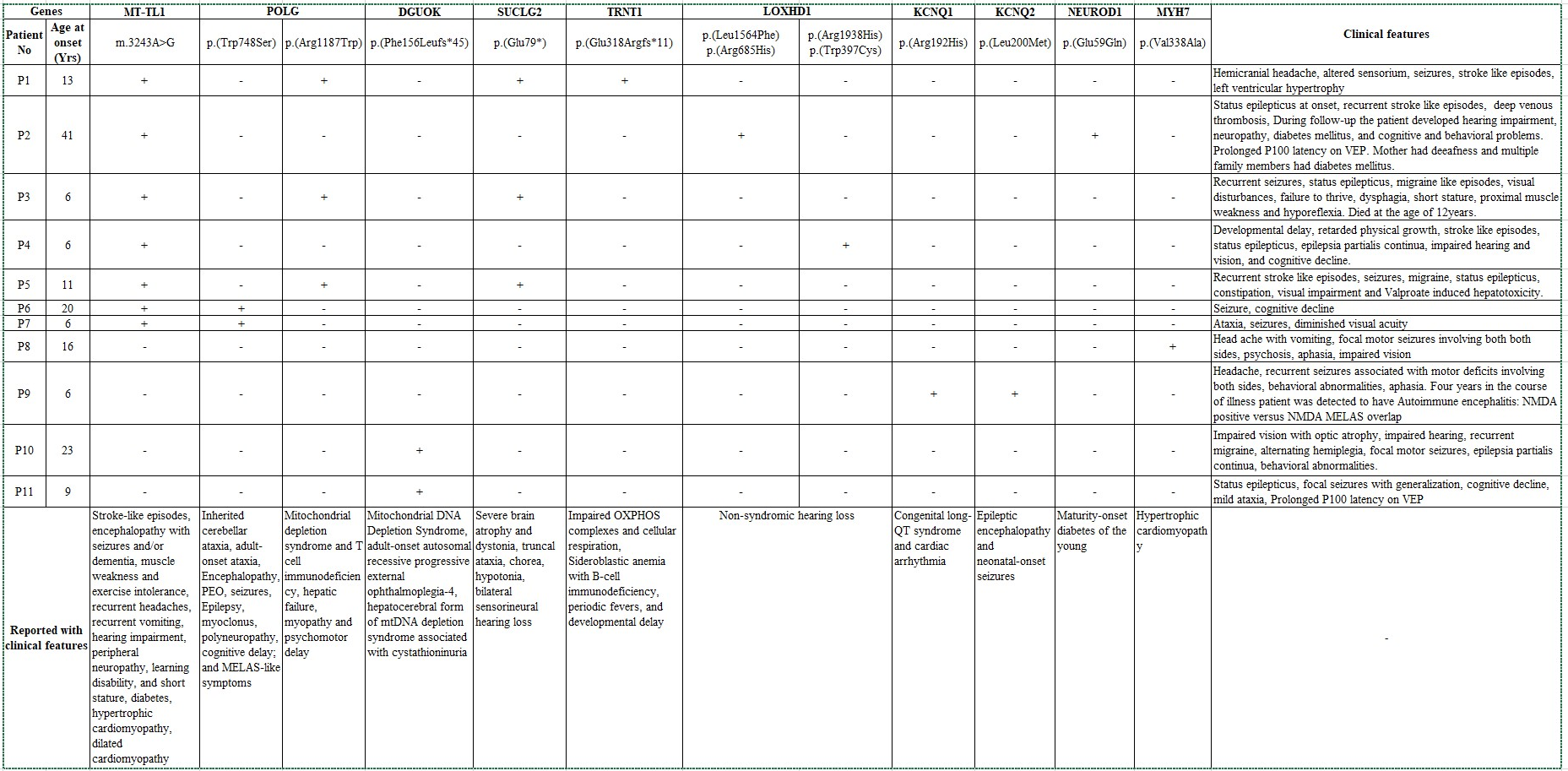
**
